# A pH-/thermo-responsive hydrogel formed from *N*,*N*′-dibenzoyl-l-cystine: properties, self-assembly structure and release behavior of SA†

**DOI:** 10.1039/c8ra09058k

**Published:** 2019-04-16

**Authors:** Jinlian Zhong, Hongyu Fu, Xinjian Jia, Haoxiang Lou, Tiantian Wan, Haiqing Luo, Huijin Liu, Dichang Zhong, Xuzhong Luo

**Affiliations:** Key Laboratory of Organo-pharmaceutical Chemistry of Jiangxi Province, Gannan Normal University Ganzhou 341000 P. R. China jiaxinjian.love@163.com zhong_dichang@hotmail.com luoxuzhong@hotmail.com

## Abstract

In this study, we report a pH-/thermo-responsive hydrogel formed by *N*,*N*′-dibenzoyl-l-cystine (DBC). It is difficult to dissolve DBC in water even on heating, and it exhibits no gelation ability. Interestingly, DBC is readily soluble in NaOH solution at room temperature and the self-assembled hydrogels are obtained by adjusting the basic DBC aqueous solution with HCl to achieve a given pH value (<3.5). When NaOH is added to the hydrogel (pH > 9.4), it becomes a sol again. This small-molecule hydrogel is characterized by scanning electron microscopy, Fourier transform infrared spectroscopy, rheological measurement and differential scanning calorimetry. The results indicate that the DBC hydrogel exhibits excellent mechanical properties, thermo-reversibility, and pH-responsive properties. Fortunately, the single crystal of DBC is obtained by volatilizing its acid aqueous solution. It crystallizes in the monoclinic space group *P*2_1_ (*Z* = 2) with lattice parameters *a* = 10.8180 (11) Å, *b* = 9.0405 (9) Å, *c* = 10.9871 (11) Å and *β* = 90.798 (3)°. By comparing the X-ray diffraction result of the DBC single crystal with that of its xerogel, the self-assembled structure of DBC in hydrogel has been ascertained. The gelators are self-assembled *via* strong intermolecular hydrogen bonds linking neighboring amide and carboxyl groups, π–π stacking interactions for aromatic rings, and hydrogen bonds between water molecules. In addition, the release behavior of salicylic acid (SA) molecules from the DBC gel is also investigated taking into account the DBC concentration, phosphate buffer solution (PBS) pH and SA concentration. When the concentrations of DBC and SA are 3.0 g L^−1^ and 200 mg L^−1^, respectively, the release ratio in PBS (pH = 4.0) reaches 58.02%. The diffusion-controlled mechanism is in accordance with Fickian diffusion control within the given time range.

## Abstract 2

A supramolecular hydrogel possessing pH-sensitive properties and thermo-reversibility was prepared, and the release behavior of salicylic acid from it is in accordance with the Fickian diffusion control within the given time.

## Graphical abstract

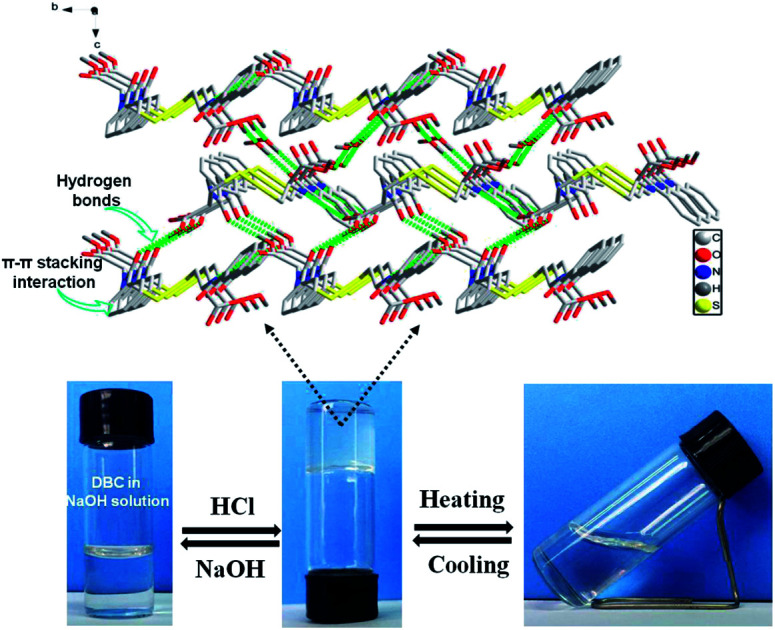

## 1. Introduction

Hydrogels have received considerable attention over the past decades because of their capabilities to entrap a large number of water molecules per gelator molecule and swelling potential, which is suitable for biomedical applications including cell culture^1^ or tissue engineering,^2–4^ controlled drug delivery systems (CDDSs),^5–9^ biosensors,^10^ and so on. As one kind of “smart” soft material, the hydrogels based on low molecular weight gelators (LMWGs) are becoming more and more familiar in the field of materials science owning to their intriguing solid-like properties.^11–13^ Generally, supramolecular hydrogels can be formed due to the self-assembly of LMWG molecules through weak non-covalent interactions,^14–17^ such as hydrogen bonding, π–π stacking, electrostatic and van der Waals interactions. They are highly attractive candidates for materials that can reversibly respond to external stimuli,^18–22^ such as pH, temperature, light, chemicals and ion.

Among the various external stimuli, the pH/temperature responsiveness of hydrogels has been most extensively investigated in sensors and biological applications due to the controllability both under *in vitro* and *in vivo* conditions.^23–25^ For example, Gao *et al.*^26^ reported a kind of softwood kraft lignin pH-responsive hydrogels which were self-assembled through strong intermolecular hydrogen bonds in aqueous solution. However, most of them are formed from polymers. Constrained synthesis, chemical cross-linking, thermosetting nature, toxicity, and slow response to external stimuli limit applications of these polymer gels. On the other hand, the “supramolecular gels” of LMWG offer various advantages over the polymer gels, such as more easily and effectively in controlling gel characteristics.^27–29^ Zhang *et al.*^30^ reported a series of aminoalkyl phosphoamide compounds that exhibited the pH-reversible gelation behavior in neutral water. The obtained hydrogels had broad application potentials in controlled drug release, recyclable catalyst carrier and so on. Although numerous multi-response gels have been reported during the last few decades, developing new pH-/temperature-responsive hydrogels from LMWG will continue to be a focus for future research.

The pH-responsive hydrogels derived from amino acid have been paid much attention because of their biocompatibility and eco-friendly nature. As an amino acid derivative, *N*,*N*′-dibenzoyl-l-cystine (DBC) consists of two amide groups and two carboxyl groups that can serve as hydrogen-bond donors and acceptors, respectively. Considering both the carboxylate and amino are able to combine with H^+^, the pH-responsive properties of DBC can be predicted. To the best of our knowledge, it is difficult to dissolve DBC in purified water even on heating. In order to improve its solubility in water, many attempts have been made to prepare the DBC hydrogel. In previous work, Gortner and Hoffman^31^ discovered that DBC could be dissolved in a beaker in 5 cm^3^ of 95% alcohol. And later, Menger and Caran^32^ extensively studied the gelation properties of DBC in DMSO/H_2_O mixtures. Additionally, Friggeri *et al.* found that DBC could gelate 1 N HCl, 1 and 5 N CH_3_COOH, aq. CaCl_2_ solution and brine.^33^ So far, however, the molecular arrangement and self-assembly structure of DBC have not been reported. Moreover, the study on the gelation properties of DBC was mainly focused on mixed-solvent systems in most examples.

Herein, we report a novel supramolecular hydrogel formed from DBC *via* adjusting solution pH. The molecular arrangement and 3D self-assembly structure of DBC in crystals was determined, and then its gelation ability was also investigated. After the morphology and microstructure of DBC gels were characterized, their pH- and thermo-responsive properties were analyzed. Moreover, the mechanical performances and self-assembly mechanism were further studied. In addition, this DBC hydrogel used for the biomedical application as the carrier of salicylic acid (SA) in controlled drug delivery systems were discussed in detail.

## 2. Experimental section

### 2.1 Materials

All chemicals were commercially available. Analytical grade DBC was purchased from J&K chemical company. Analytical grade SA, NaOH, HCl, citric acid, Na_2_HPO_4_, AgNO_3_, NaCl and KH_2_PO_4_ were purchased from Tianjin great chemical reagent factory. Phosphate buffer solutions (PBS, pH = 4.0 and 7.4) were prepared by a standard method.

### 2.2 Gelation test

The gelation ability of DBC was investigated by a typical test tube experiment. The weighed hydrogelator DBC was mixed with 1.0 mL of NaOH solution (mol ratio = 1 : 2) in a test-tube with a cap until forming a clear solution. The resulting solution was adjusted to a given pH (<3.5) with HCl at room temperature to obtain a stable hydrogel, which was confirmed by inverting the test-tube containing the solution. A required minimum amount of DBC for gelation was defined as the minimum gelator concentration (MGC).

### 2.3 Production of freeze-dried hydrogels

In a typical experiment, a certain amount of water was carefully placed on top of the DBC hydrogel in a beaker. Subsequently, the exchange between water and NaCl molecules within the DBC gel occured. The test was carried out several times. Finally, AgNO_3_ solution was used for the detection of Cl^−^ within the gel. After NaCl was completely exchanged by water molecules, the beaker containing DBC hydrogels was transferred into a freeze-drying vessel (FD-1-50, Beijing BoYiKang Experimental Instrument Co., Ltd.) and dried at −50 °C for 24 h under vacuum condition (<100 pa).

### 2.4 Entrapment and release of SA from supramolecular hydrogels

A certain amount of DBC and SA were dissolved into ethanol with continuous stirring. Subsequently, the solution was added to a test-tube containing the PBS with a defined pH, forming stable hydrogel. The calibration curve was obtained by the gradually dilution of SA aqueous solutions (100 mg L^−1^) and measuring its maximum absorbance at 297 nm by using a UV-Vis spectrophotometer. The curves showed excellent linear relationships between maximum absorbance and concentration in the range of 0–100 mg L^−1^. The maximum absorbance wavelength of DBC was found to be at 226 nm.

For the release experiment, 8 mL of various pH buffer solutions used as the SA receiving media were carefully placed on top of each hydrogel in a test-tube. At the given interval, 3 mL of supernatant solution was taken out. Meanwhile, 3 mL of buffer solution were added to the test-tube for maintaining balance. The absorbance at 297 nm for each sample was measured, and the concentration of SA that has been released from the hydrogel was obtained based on the calibration curves. All experiments were carried out in triplicate.

### 2.5 Characterization

Scanning electron microscopy (SEM) images were captured using a FEI QUANTA 450 with an accelerating voltage of 15.0 kV. The preparation of samples for SEM involved placing a drop of hydrogel on the copper substrate. The hydrogel was subjected to shock-freezing by liquid nitrogen, followed by lyophilization for 3 h. And then, it was submitted to a SEM scan after gold-coating for 3 min. Fourier transform infrared (FT-IR) spectra measurements were carried out on a Nicolet iS50 FT-IR spectrophotometer. CaF_2_ substrates and KBr pellets were used for transmission spectra of xerogel and powder, respectively. A drop of hydrogel was casted onto CaF_2_ substrates dried at 60 °C by vacuumizing method, and then the obtained xerogel was subjected to FT-IR spectra measurements. X-ray powder diffraction (XRD) patterns of dried hydrogels (xerogels) were collected on a Bruker D8 Focus diffractometer with the Cu-Kα radiation (*λ* = 1.5418 Å). The methods for preparing the dried hydrogels were shown in the ESI.† Single-crystal data were collected on a Bruker Smart APEX II diffractometer, with Mo-Kα radiation (*λ* = 0.71073 Å). Differential scanning calorimetry (DSC) was conducted on Setaram μDSC7-Evo instrument (heating rate, 1 °C min^−1^). UV absorption spectra were obtained using a UV-1800 UV-Vis spectrophotometer. Rheological measurement was performed by using a stress-controlled rheometer (HAAKE RheoStress 6000) with parallel plate type geometry (plate diameter, 3.5 cm). A solvent trap equipped with the rheometer was used to protect the sample from evaporation. Temperature dependences of the storage shear moduli (*G*′) and the loss shear moduli (*G*′′) were carried out by heating the sample at a rate of 0.1 °C s^−1^. The viscoelastic moduli was monitored under small-amplitude oscillatory shear at an applied frequency of 1 Hz and stress of 1 Pa. Frequency sweeps at selected temperatures were carried out over a range of 0.1–100 rad s^−1^ at a stress of 1 Pa.

## 3. Results and discussion

### 3.1 Gelation properties

**Fig. 1:**
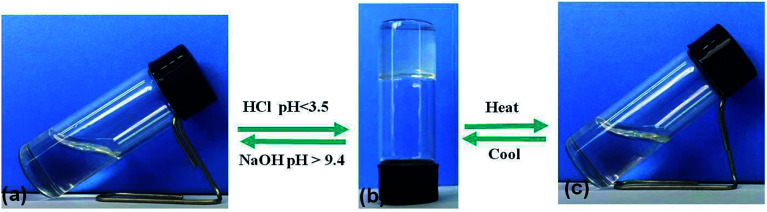
Schematic diagram of the gel–sol transition process: (a) DBC dissolved in NaOH solution; (b) the obtained hydrogel after adding HCl solution; (c) the DBC sol after heating.

According to the “gelation test” method (Section 2.2), the gelation ability of compound DBC in water was investigated. We discovered that DBC was readily soluble in NaOH solution at room temperature. And then, a stable transparent hydrogel was obtained by adjusting the solution pH to a given value (<3.5). The formed gels can maintain gelation for over 1 month. Intriguingly, by adding NaOH to the gel, it returns to the sol state again (pH > 9.4), indicating that this DBC hydrogel exhibits pH-sensitive properties (Fig. 1a and b). In addition, the DBC hydrogel can also be transformed into a sol upon heating. Subsequently, it self-assembles into a transparent gel again after cooling down to room temperature slowly. The gelation process is repeatable (Fig. 1b and c). These results indicate that the DBC hydrogel possesses both pH-responsive property and thermo-reversibility. In order to determine the gelatinizing ability of DBC, its MGC is measured. The result is 1.5 g L^−1^, a low MGC values at room temperature, indicating that DBC is a highly efficient hydro-gelator.^34^

**Fig. 2:**
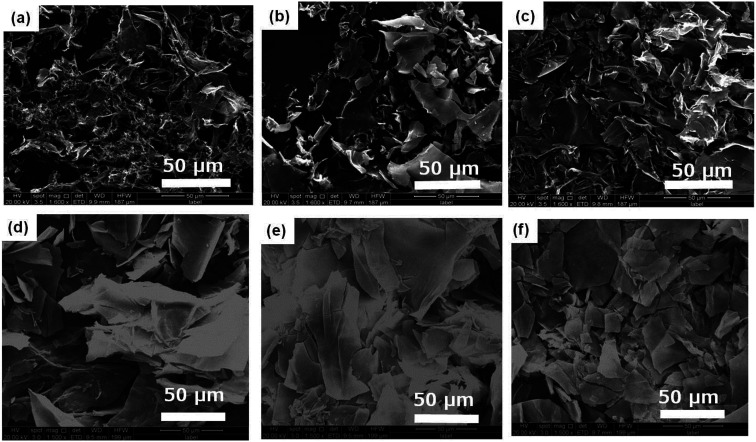
SEM images of DBC hydrogels formed by varying concentrations: (a) 1.5 g L^−1^; (b) 2.0 g L^−1^; (c) 3.0 g L^−1^; (d) 4.0 g L^−1^; (e) 5.0 g L^−1^; (f) 6.0 g L^−1^.

The aggregation morphologies of DBC xerogels were observed using SEM. All samples are prepared by freeze-drying for avoiding the damage from high vacuum or drying. The corresponding images are shown in Fig. 2 and S1.† From the SEM images of DBC xerogels, we can find that sheet-like structure with the width of 5–20 μm at low concentrations is formed. Moreover, similar sheet-like structures with the width of 10–50 μm at high concentrations (7.0, 8.0, 9.0, 10.0 g L^−1^) are also observed. Gelator molecules creates complex three-dimensional networks by entangling numerous sheet-like structures and entraps abundant water in the interspace of the networks by surface tension and capillary forces, leading to the formation of DBC hydrogels.^35^

**Fig. 3:**
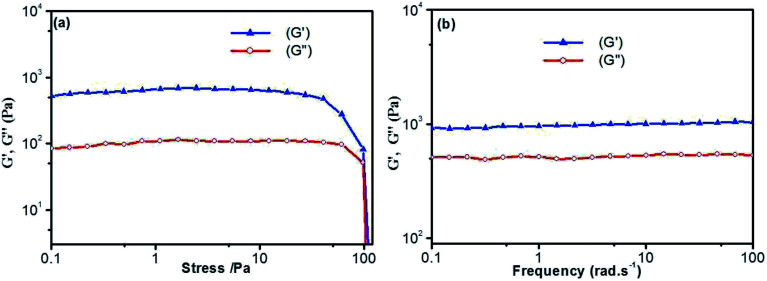
Dynamic rheology of hydrogel (4.0 g L^−1^): (a) stress sweep; (b) frequency sweep.

**Fig. 4:**
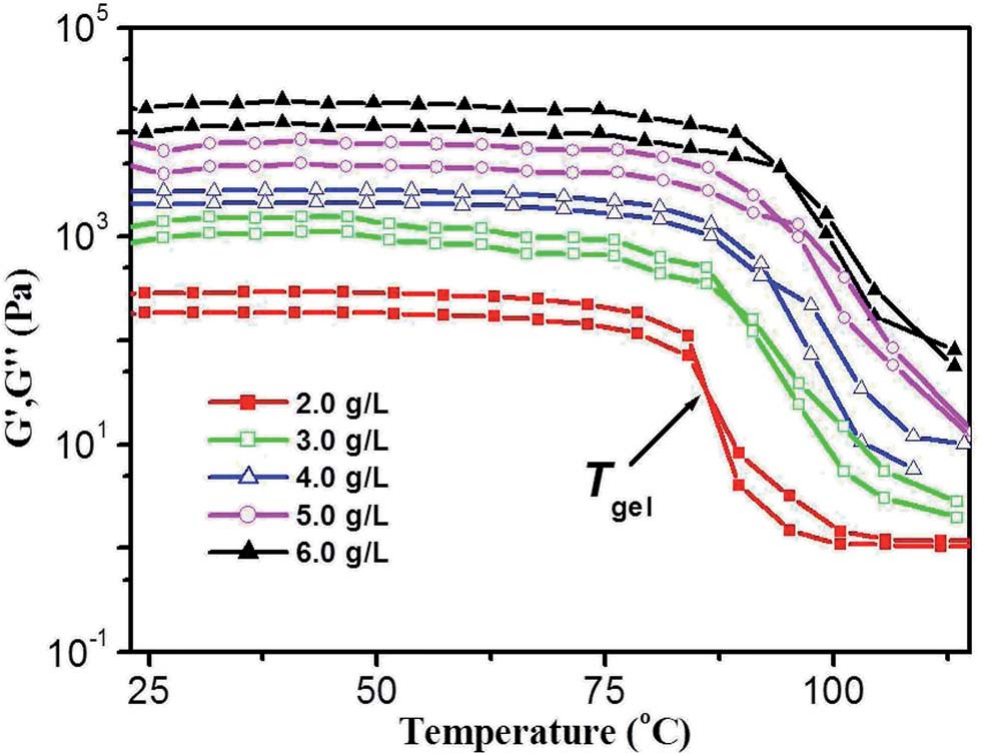
Temperature dependence of *G*′ and *G*′′ of DBC gel at varying concentrations. (Gelator concentrations: 2.0 g L^−1^, 3.0 g L^−1^, 4.0 g L^−1^, 5.0 g L^−1^ and 6.0 g L^−1^.)

Thermal stability and thermo-reversibility of the gel are interesting in respect to its various applications, such as drug delivery.^36^ In order to evaluate the thermal stability of DBC gels, rheological and DSC measurements were conducted.^37^ Generally speaking, hydrogels start to flow at a shear stress when they succumb to an applied stress. According to the stress sweep (Fig. 3a and S2 in ESI†), the value of the storage modulus (*G*′) for DBC hydrogels is much higher than that of the loss modulus (*G*′′). Below a certain level of stress, *G*′ and *G*′′ are independent of the stress, and the deformation is always close to 0, inferring that the gel structure keeps completely intact.^38^ Beyond a certain level of stress, a catastrophic disruption of the gels occurs, as indicated by a steep drop in the values of both moduli and the reversal of the viscoelastic signal. As *G*′ and *G*′′ drop sharply, the gels are deformed to a certain extent. At room temperature, the frequency sweep exhibits typical solid-like rheological behavior with *G*′ dominating *G*′′ over the investigated oscillating frequency range (Fig. 3b and S3 in ESI†). As shown in Fig. 3b, the *G*′ and *G*′′ of DBC gels formed by varying concentration slightly increase upon increasing the frequency from 0.01 to 100 Hz. Moreover, the value of *G*′ is always larger than that of *G*′′ in the whole range (0.01–100 Hz), suggesting that the gels are fairly tolerant to the external force and are effective physical gels. Additionally, the temperature dependences of *G*′ and *G*′′ are also conducted to characterize the thermo-responsive behaviors of DBC gels.^39^ Temperature measured by rheology experiments in which *G*′ = *G*′′ is denoted as *T*_gel_.^40^ As depicted in Fig. 4, both *G*′ and *G*′′ almost keep constant at low temperature but decrease rapidly at *T*_gel_. For instance, both *G*′ and *G*′′ of the DBC gel at 2.0 g L^−1^ almost keep constant from room temperature to 82.6 °C, above which they decrease rapidly with the increase of temperature, indicating the gradual transformation from gel to sol. When the temperature reaches 85.5 °C, a cross-over point where *G*′ equals to *G*′′ appears, indicating the transition from primarily elastic to viscous properties. Upon further heating to 89.5 °C, *G*′′ exceeds *G*′, implying that the gel is transformed to sol completely. Similar phenomenons are also observed for DBC gel at 3.0, 4.0, 5.0 and 6.0 g L^−1^, and the corresponding *T*_gel_ is 88.7, 92.2, 92.3 and 92.5 °C, respectively. These results demonstrate that the stability of hydrogel is gradually enhanced on increasing the DBC concentration. However, when the concentration is increased to a certain value, *T*_gel_ levels off. Similar results are also obtained by DSC experiments as follows.

**Fig. 5:**
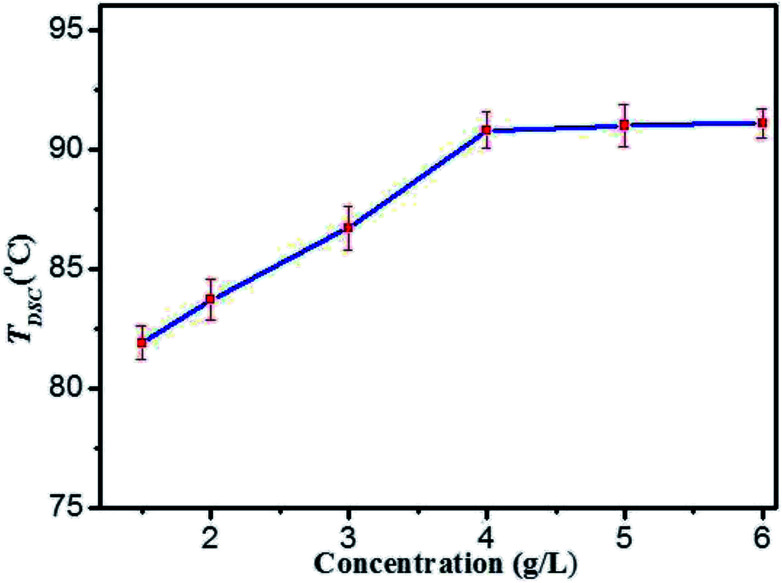
The gel–sol transition temperatures (*T*_DSC_) profiles of DBC hydrogels prepared by varying concentrations.

On the basis of rheological results, DSC measurements provide further insight into the thermo-reversibility in detail. Typical heating and cooling DSC curves of DBC hydrogels are presented (Fig. S4 in ESI†), and the temperature corresponding to the maximum of the peak in DSC curves is denoted as *T*_DSC_.^40^ By visual inspection, the samples are liquids above *T*_DSC_, and then become into gels below *T*_DSC_. The *T*_DSC_ profiles of DBC hydrogels prepared by varying concentrations (Fig. 5) illustrate that it gradually increases with increasing the DBC concentration and is virtually independent of concentration to 4.0 g L^−1^. This phenomenon is consistent with that obtained by the rheology experiments. Consequently, both mechanical and thermal experimental results clearly display two trends: an increase at low concentrations and a plateau above a threshold concentration within the selected temperature range. The probable explanation is that incomplete aggregate network is gradually becoming complete upon increasing the DBC concentration from 1.5 to 4.0 g L^−1^. Hence, a high temperature is needed to break the aggregate structure.^41,42^ When the concentration reaches 4.0 g L^−1^, complete aggregate network is formed and additional DBC serves a secondary role to stabilize the internal structure of the hydrogel. Therefore, *T*_DSC_ increases only slightly when DBC is added over the certain concentration.

### 3.2 Molecular arrangement and self-assembly mechanism

**Fig. 6:**
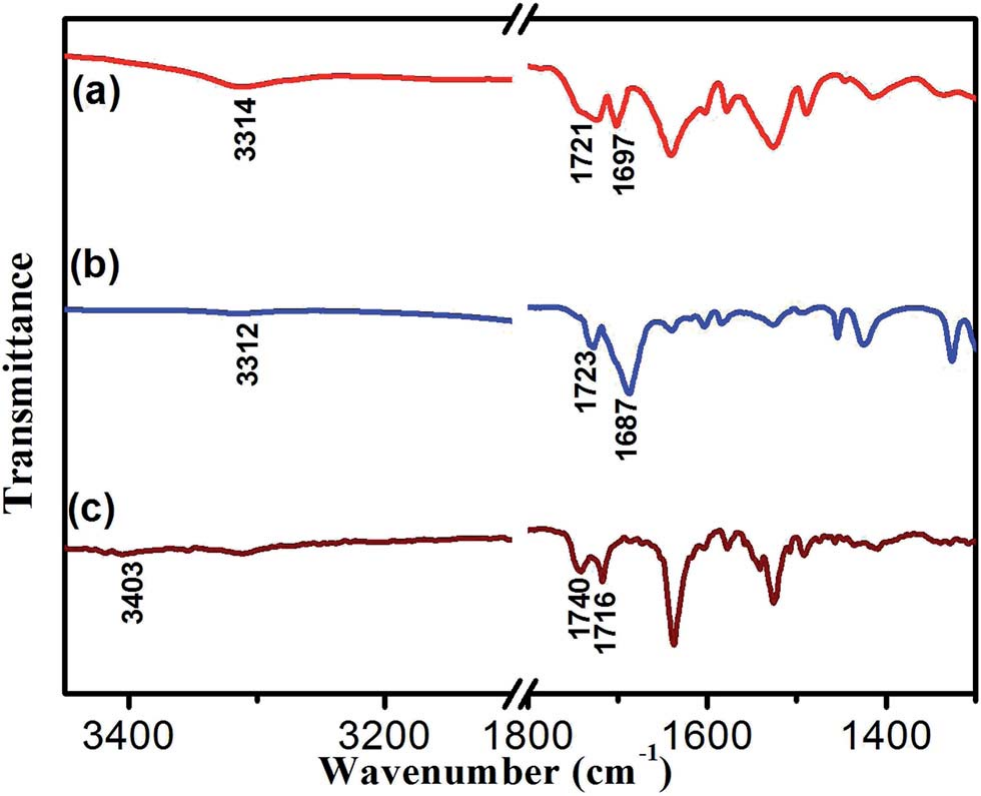
FT-IR spectra of (a) DBC crystal, (b) xerogel and (c) DBC in CCl_4_ solution.

**Fig. 7:**
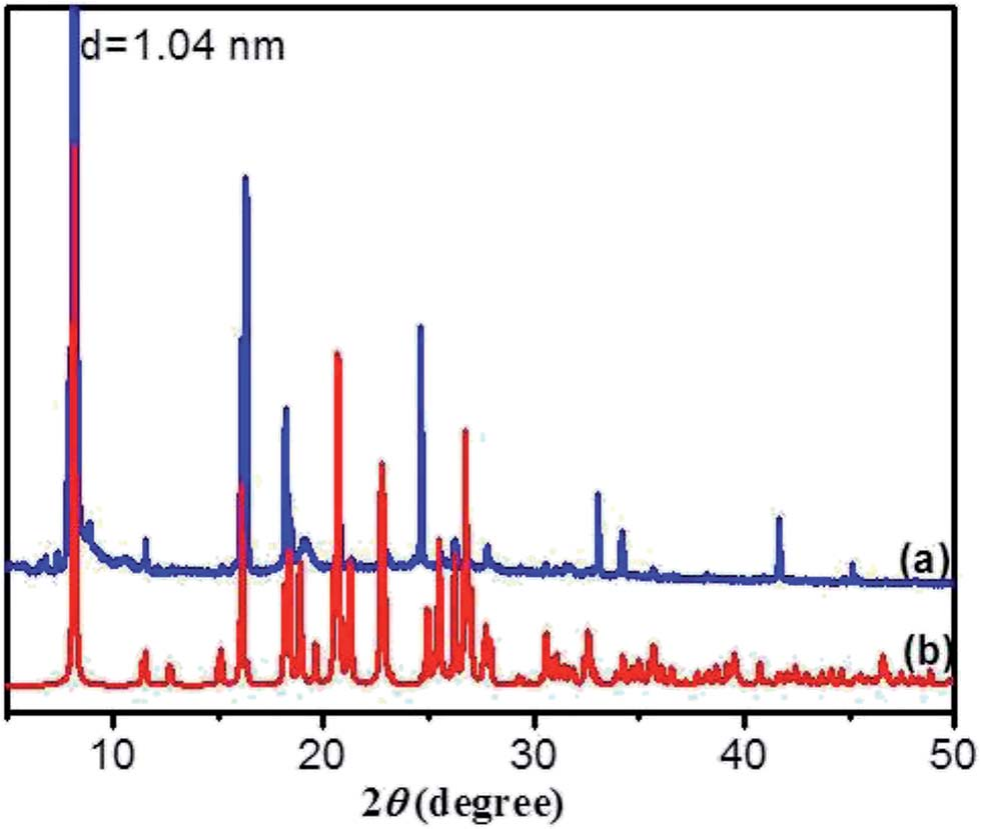
XRD patterns of (a) DBC xerogel and (b) its crystal.

To reveal the molecular interaction and aggregative structure of DBC hydrogel, FT-IR and XRD analyses have been conducted. The information of the hydrogen-bonding environment for amide groups can be sensitively detected by FT-IR spectroscopy. For the DBC xerogel (Fig. 6b), the bands that appear at 3312 and 1687 cm^−1^ can be attributed to the *ν*N–H and *ν*C=O for amide groups, respectively. Compared to the N–H stretching band of the free secondary amide group (3403 cm^−1^, Fig. 6c), a red shift is found for that of the DBC xerogel, suggesting that hydrogen bonds are formed between neighboring amides.^43–46^ Additionally, the band corresponding to the C=O stretching vibration of the carboxyl group occurs a red-shift from 1740 cm^−1^ (free *ν*C=O of carboxylic acid) to 1723 cm^−1^, implying that the C=O in carboxyl groups takes part in the formation of intermolecular hydrogen bonds. Moreover, the corresponding vibration bands of the crystal (Fig. 6a) also undergo a red shift, which is similar to that of the xerogel, indicating that the pattern of hydrogen bonding in crystal is close to that in gel. These results reveal that there are intermolecular hydrogen bonds between neighboring amide groups and carboxyl groups, which are juxtaposed and interlocked by van der Waals interaction and finally gelate the water molecules. Generally, X-ray crystallography is a powerful technique for characterizing the structures of various types of colloidal dispersions.^47^ The aggregation structure of the DBC xerogel was confirmed by XRD. Fig. 7 shows the XRD patterns of the DBC xerogel and its crystal. Compared with the DBC crystal, the diffraction peaks at 2*θ* = 8.2°, 11.5°, 16.3°, 18.2°, 20.8°, 24.6° and 26.3° are appeared in the same position for its xerogel, and there are no new peak observed. Therefore, it can be inferred that both the DBC crystal and its hydrogel have similar assembly structures.

**Fig. 8:**
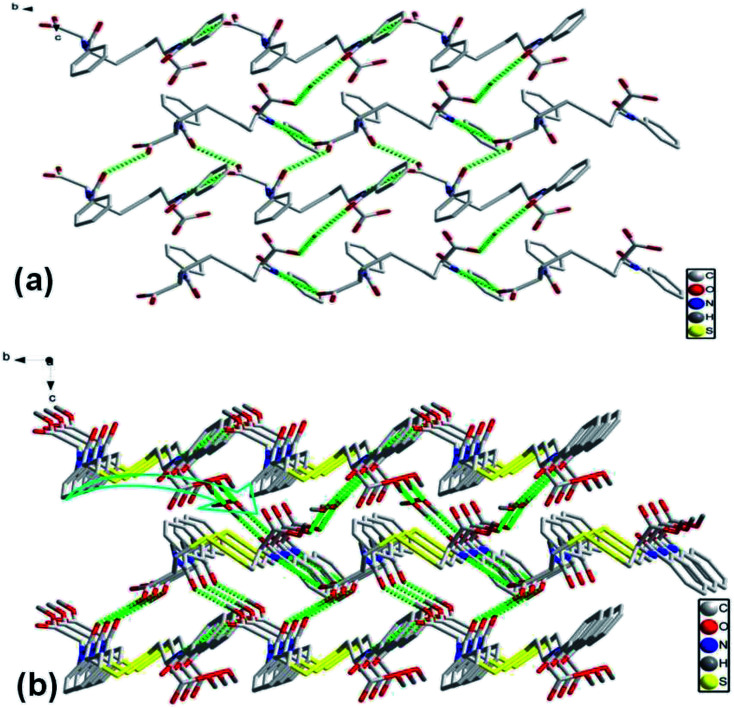
The self-assembly structure of DBC crystal: (a) top-view of the 2D sheet and (b) 3D crystal structure.

As crystallized in acid aqueous solution, the DBC crystal that is suitable for single crystal X-ray diffraction analysis was obtained. In order to understand the molecular arrangement of DBC crystal, we decided to investigate its single crystal structure. The crystal data, selected bond lengths and angles are listed in Tables S1 and S2, respectively (ESI†). Further details about the structure information have been deposited at the Cambridge Crystallographic Data Centre (CCDC) as supplementary publication CCDC 1870748. As illustrated in Table S1 (ESI),† DBC crystallizes in the monoclinic space group *P*2_1_ (*Z* = 2) with lattice parameters *a* = 10.8180 (11) Å, *b* = 9.0405 (9) Å, *c* = 10.9871 (11) Å and *β* = 90.798 (3)°. As displayed in Fig. 8, there are rich hydrogen bond interactions in water and DBC molecules, including O7W–H⋯O1 (O7W⋯O1, 2.6524 Å), O7W–H⋯O2 (O7W⋯O2, 2.5896 Å), N2–H⋯O3 (N2⋯O3, 2.9868 Å), N1–H⋯O5 (N1⋯O5, 2.9221 Å) and O4–H⋯O6 (O4⋯O6, 2.6477 Å). Each DBC molecule connects with another six DBC molecules *via* eight intermolecular hydrogen bonds to form a 2D supermolecular layer (Fig. 8a). The thickness of this layer measured from the crystal structure is 1.02 nm, which is in close proximity to 1.04 nm, the long spacing of the DBC gel (Fig. 7a). Each layer is further linked with the adjacent layers through π–π stacking interactions between aromatic rings, generating a 3D supermolecular structure (Fig. 8b).

### 3.3 Release behavior of DBC hydrogels

**Fig. 9:**
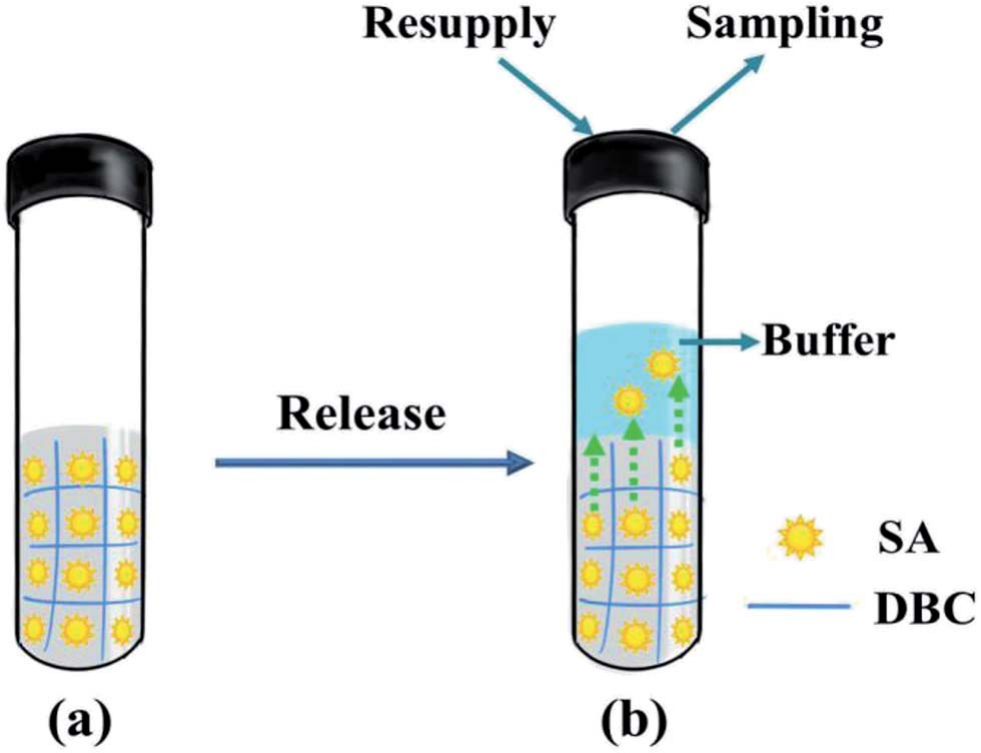
Schematic depiction for the release of SA from the DBC gel: (a) the DBC hydrogel containing SA molecules; (b) the release of SA from the as-prepared hydrogel.

Owing to the solid-like properties of supramolecular hydrogels formed from LMWGs, they possess potential practical applications in the field of vehicles for controlled drug release.^48^ Herein, a study concerning DBC hydrogels as controlled release systems for SA molecules was presented, and the corresponding release behavior was also investigated in detail. The release process of SA from the DBC hydrogel is illustrated in Fig. 9.

**Fig. 10:**
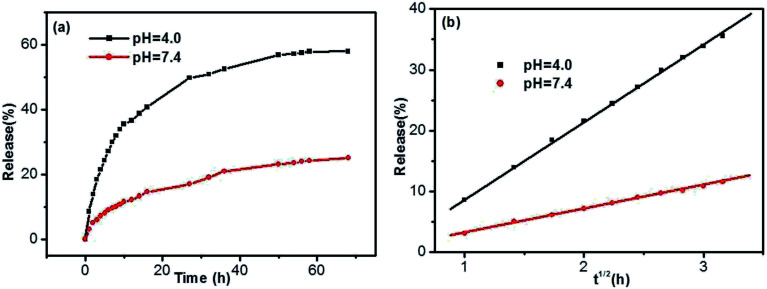
Release (a) ratios and (b) kinetics of SA from the hydrogels formed by 3.0 g L^−1^ of DBC in the case of various pH buffer solutions as receptor (initial SA concentration: 200 mg L^−1^).

Taking into account the important role of solution pH in release processes, the SA release from the DBC hydrogel at different PBS pH was investigated. As shown in Fig. 10, the release ratios of SA are various when PBS with different pH is used as SA receptors. In comparison with the pH of 7.4, the release ratio of SA for the pH of 4.0 is higher (Fig. 10a). In other words, the acidic receiving medium for SA release from the DBC hydrogel is more effective than the neutral receiving medium. This can be attributed to the variation of supramolecular interactions in the gels at different pH conditions.^49^ Somewhat interestingly, when the amount of SA released from the DBC hydrogel is plotted against the square root of time (within 10 h), a good linear correlation is displayed (Fig. 10b). Usually, Korsmeyer–Peppas model (*M*_*t*_/*M*_∞_ = *kt*^*n*^) can be applied to evaluate the relationship between release rate and time.^50^ When *n* = 0.5, the release process is in according with Fickian diffusion control. For 0.5 < *n* < 1.0, it is consistent with anomalous (non-Fickian) diffusion control. In this release process of SA from the DBC hydrogels, the value of “*n*” is 0.5, implying that the process is in accord with Fickian diffusion mechanism within the given time range.^51^

**Fig. 11:**
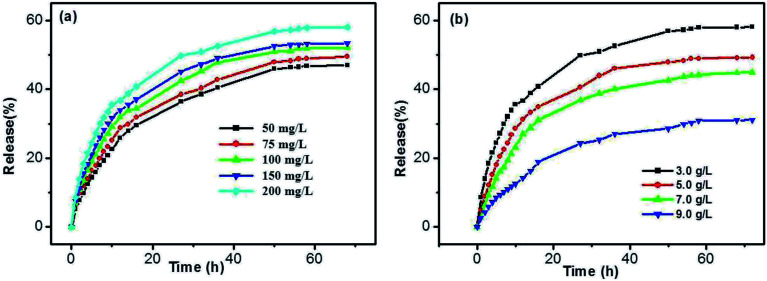
Effects of (a) SA and (b) DBC concentrations on the release ratio at 25 °C.

In addition, the influences of SA and DBC concentrations were also investigated. As shown in Fig. 11a, the release rates of SA gradually increase for all samples upon increasing the SA concentration. There is no significant difference in the case of low concentrations for SA, such as in 50 and 75 mg L^−1^. However, the release rates are faster in the case of high concentrations for SA (such as in 150 and 200 mg L^−1^). Generally, the rate of diffusion-controlled release only depends on the solute concentration difference at both sides of the hydrogel/solution interface, and not on the solute concentration within the hydrogel. In the DBC hydrogels, SA molecules are entrapped in the sheet-like structure. As the increase of the SA concentration, sheet-like structure fabricated by the DBC hydrogelator may be disrupted. Thereby, 3D networks within the hydrogel tend to partial collapse, leading to an increase in the released amount of SA. As depicted in Fig. 11b, a typical sustained release behavior that the release ratio of SA from the hydrogels decreases with the increase of the DBC concentration is obviously observed. The max release ratios of SA are 31.01%, 44.85%, 49.15% and 58.02% when the DBC concentrations are 9.0, 7.0, 5.0 and 3.0 g L^−1^, respectively. This phenomenon can be explained by assuming that the low release rate of SA is related to the presence of dense 3D networks formed by increasing the DBC concentration.^52^ Interestingly, when the amount of SA released from the hydrogel is plotted against the square root of time (Fig. S5 in ESI†), the corresponding linear relationship also exists. It indicates that the release mechanism of SA from the hydrogels also follows the Fickian diffusion control before 10 h. Based on these results, it can be deduced that the release ratio of SA in the DBC hydrogels (3.0 g L^−1^) is optimal at high concentrations of SA and the pH of 4.0.

## 4. Conclusions

In summary, a novel pH/thermo-responsive hydrogel formed from DBC is prepared, and the corresponding single crystal is also obtained. The structural investigation reveals that DBC molecules are self-assembled by strong intermolecular hydrogen bonds linking neighboring amides and carboxylic acid, π–π stacking interactions for aromatic rings, and hydrogen bonds between water molecules. Moreover, the as-prepared hydrogels possess good mechanical property, pH-sensitiveness and thermo-reversibility. Meanwhile, they also display the potential in drug delivery applications as the carrier of SA. The concentrations of DBC and SA, as well as the pH of PBS can all affect the release ratio of SA. The optimal release condition of SA in DBC hydrogels (3.0 g L^−1^) is at high SA concentrations for the pH of 4.0. The release mechanism is in accordance with Fickian diffusion control within the given time range. In addition to the application in controlled drug release system, this kind of DBC hydrogel is expected to be used as a new pH-responsive material.

## Conflicts of interest

There are no conflicts of interest to declare.

## Supplementary Material

RA-009-C8RA09058K-s001

RA-009-C8RA09058K-s002
