# Supplementary material for: A pH-/thermo-responsive hydrogel formed from N,N′-dibenzoyl-l-cystine: properties, self-assembly structure and release behavior of SA
Source: RSC Adv. 2019 Apr 16;9(21):11824–32. doi: 10.1039/c8ra09058k (PMC9063318; doi:10.1039/c8ra09058k)
Supplement: RA-009-C8RA09058K-s001 [file RA-009-C8RA09058K-s001.pdf]

Electronic Supplementary Material (ESI) for RSC Advances.
This journal is © The Royal Society of Chemistry 2019

Electronic Supplementary Information (ESI)

A pH-/thermo-responsive hydrogel formed from *N,N′*-dibenzoyl-L-cystine: property, self-assembly structure and release behavior of SA

Jinlian Zhong, Hongyu Fu, Xinjian Jia, Haoxiang Lou, Tiantian Wan, Haiqing Luo, Huijin Liu, Dichang Zhong*, Xuzhong Luo*

Key Laboratory of Organo-Pharmaceutical Chemistry of Jiangxi Province, Gannan Normal University, Ganzhou, 341000, China. E-mail: luoxuzhong@hotmail.com; zhong_dichang@hotmail.com

1. Synthesis of xerogels

After the DBC gel containing NaCl was completely exchanged by water molecules, the corresponding DBC xerogel was obtained by drying the gel in a vacuum oven at 60 ^{o}C for 24 h.

2. Growth of DBC Crystal

First, 10.0 mg of DBC was mixed with 5 mL of the NaOH solution (0.5 mg/L) until the solid was completely dissolved. And then, the mixture was adjusted to a given pH with aqueous HCl solutions until obtaining a stable hydrogel at ambient temperature. After five days, colorless crystals suitable for single-crystal X-ray diffraction analysis were finally obtained by filtration.

3. SEM

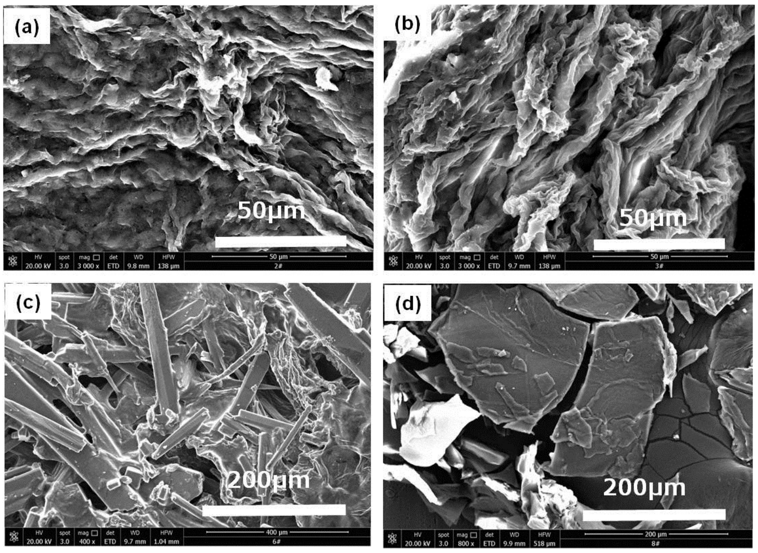

Fig. S1 SEM images of DBC hydrogels: (a)7.0 g/L; (a)8.0 g/L; (a)9.0 g/L; (d)10.0 g/L

4. Rheological behavior of hydrogels

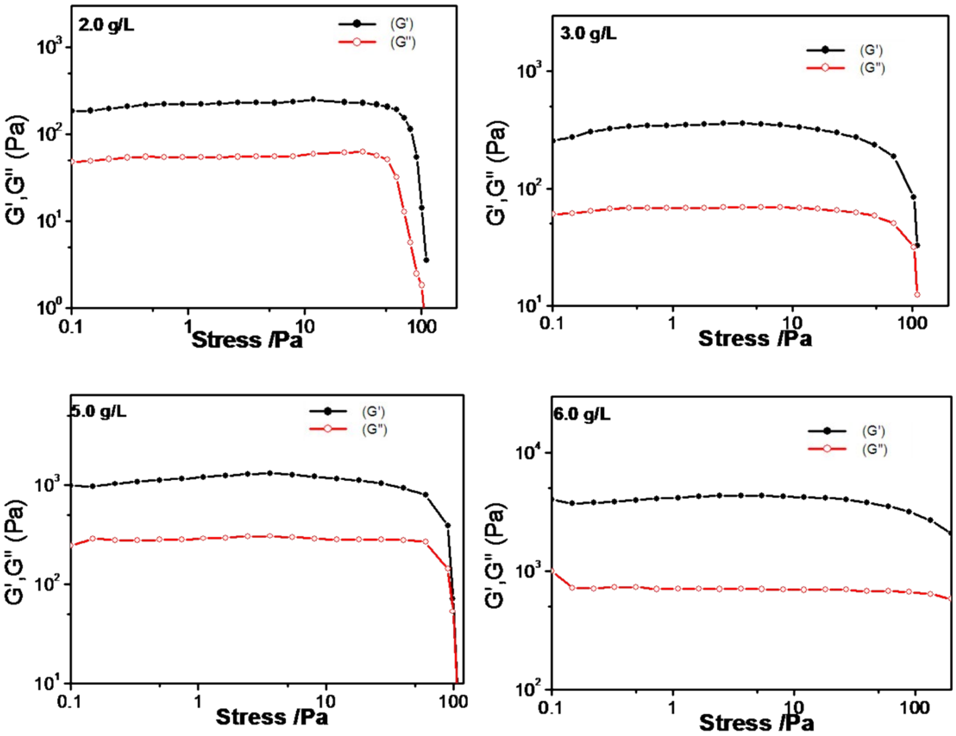

Fig. S2 The stress sweep of DBC gel formed by varying concentration (2.0 g/L, 3.0 g/L, 5.0 g/L, 6.0 g/L)

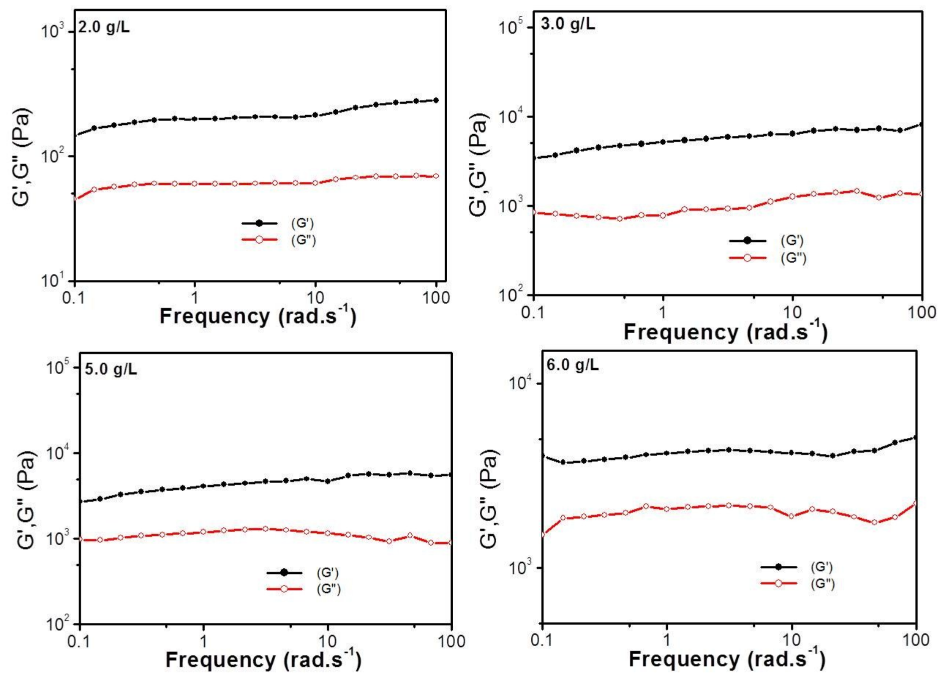

Fig. S3 Rheological measurements of DBC hydrogels with the mode of frequency sweep for *G′* and *G″* (gelator concentrations: 2.0 g/L, 3.0 g/L, 5.0 g/L and 6.0 g/L).

5. Date of differential scanning calorimetry (DSC)

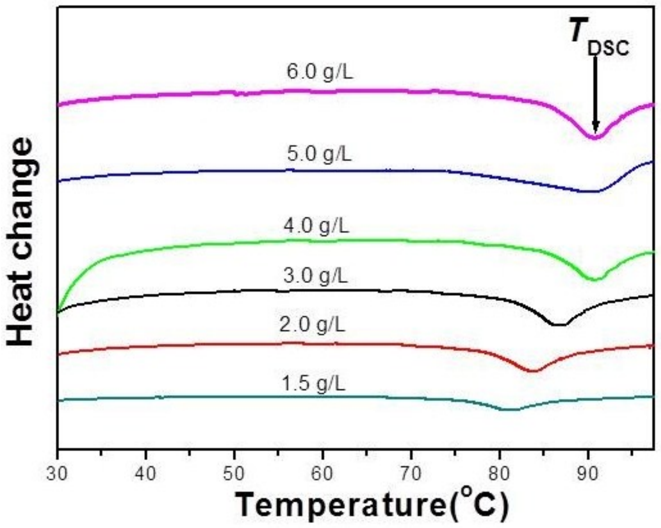

Fig. S4 DSC thermograms of DBC hydrogels prepared at various gelator concentrations (gelator concentrations: 1.5 g/L, 2.0 g/L, 3.0 g/L, 4.0 g/L, 5.0 g/L and 6.0 g/L).

6. Structure of DBC Crystal

Table S1. Crystal data and structure refinement for DBC.

Compound	DBC
Formula	$C_{20}H_{20}N_2O_7S_2$
$F_{W.}$	464.5
Crystal system	Monoclinic
Space group	$P2_1$
a (Å)	10.8180(11)
b (Å)	9.0405(9)
c (Å)	10.9871(11)
α (˚)	90
β (˚)	90.798(3)
γ (˚)	90
V (Å^3)	1074.44(19)
Z	2
D_c (g·cm^{-3})	1.436
Reflections/ unique	5104/4073
$R_{(int)}$	0.0388
GOF on F^2	1.043
$R_1[I \geq 2\sigma(I)]$	0.0472
$wR_2[I \geq 2\sigma(I)]$	0.1149

Table S2. Selected bond distances (Å) and angles (º) for DBC.

C13—O5	1.198(3)	C10—S2	1.808(3)
C13—O4	1.322(3)	C3—C2	1.354(4)
C13—C12	1.514(3)	C3—C4	1.364(4)
O6—C14	1.228(3)	C20—C19	1.378(4)
N2—C14	1.334(3)	C9—O3	1.197(3)
N2—C12	1.439(3)	C9—O2	1.311(3)
C14—C15	1.494(3)	C5—C6	1.373(4)
N1—C7	1.329(3)	C5—C4	1.377(4)
N1—C8	1.451(3)	C17—C18	1.370(5)
C15—C16	1.382(4)	C1—C2	1.378(4)
C15—C20	1.388(4)	C1—C6	1.390(4)
C16—C17	1.384(4)	C18—C19	1.358(5)
C7—O1	1.224(3)	C11—S1	1.809(2)
C7—C6	1.483(4)	S1—S2	2.0231(9)
C8—C9	1.516(3)	O1—O7Wi	7.6384(39)
O5—C13—O4	123.4(2)	C13—C12—C11	111.24(18)
O5—C13—C12	124.3(2)	C8—C10—S2	115.79(16)
O4—C13—C12	112.2(2)	C2—C3—C4	119.4(3)
C14—N2—C12	122.1(2)	C19—C20—C15	119.8(3)
O6—C14—N2	121.3(2)	O3—C9—O2	125.4(2)
O6—C14—C15	121.8(2)	O3—C9—C8	122.3(2)
N2—C14—C15	116.9(2)	O2—C9—C8	112.3(2)
C7—N1—C8	124.1(2)	C6—C5—C4	120.6(2)
C16—C15—C20	119.3(2)	C18—C17—C16	120.5(3)
C16—C15—C14	123.1(2)	C3—C4—C5	120.7(3)
C20—C15—C14	117.6(2)	C2—C1—C6	120.3(3)
C15—C16—C17	119.7(3)	C19—C18—C17	120.0(3)
O1—C7—N1	121.4(3)	C3—C2—C1	120.8(3)

O1—C7—C6	120.2(2)	C5—C6—C1	118.1(2)
N1—C7—C6	118.3(2)	C5—C6—C7	123.9(2)
N1—C8—C9	113.1(2)	C1—C6—C7	118.0(2)
N1—C8—C10	107.99(19)	C18—C19—C20	120.8(3)
C9—C8—C10	110.7(2)	C12—C11—S1	114.52(16)
N2—C12—C13	110.41(18)	C11—S1—S2	104.43(8)
N2—C12—C11	111.17(18)	C10—S2—S1	105.21(9

7. Release behavior of DBC hydrogel

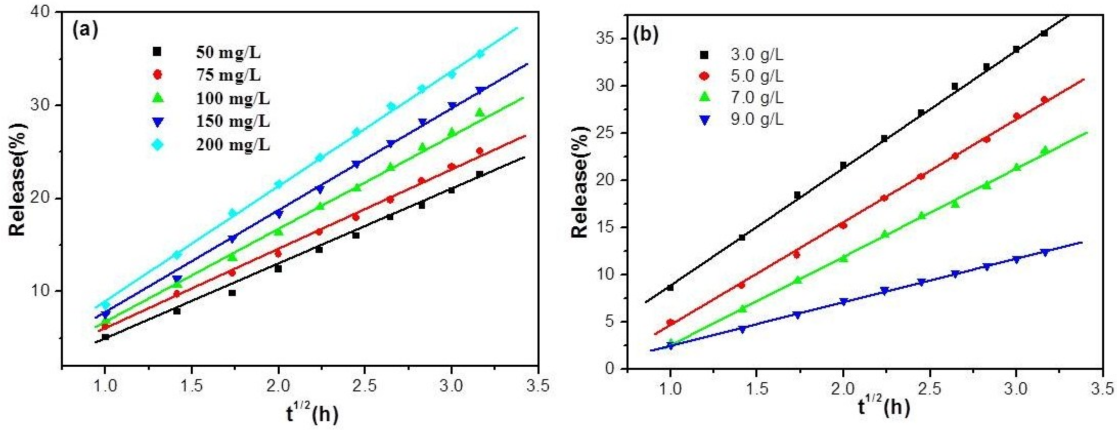

Fig. S5 Release kinetics: (a) different SA concentrations from the DBC hydrogels (3.0 g/L) at 25 ^{o}C; (b) SA from the DBC hydrogels formed by different concentrations at 25 ^{o}C.
